# Editorial: Microorganism and process engineering for biosynthesis

**DOI:** 10.3389/fbioe.2023.1239991

**Published:** 2023-07-03

**Authors:** Xianghui Qi, Jiandong Cui, M. Mofijur, Hossain M. Zabed

**Affiliations:** ^1^ School of Life Sciences, Guangzhou University, Guangzhou, Guangdong, China; ^2^ School of Food and Biological Engineering, Jiangsu University, Zhenjiang, Jiangsu, China; ^3^ State Key Laboratory of Food Nutrition and Safety, Laboratory of Industrial Fermentation Microbiology, Ministry of Education, Tianjin University of Science and Technology, Tianjin Economic and Technological Development Area (TEDA), Tianjin, China; ^4^ Faculty of Engineering and IT, University of Technology Sydney, Sydney, NSW, Australia; ^5^ Mechanical Engineering Department, Prince Mohammad Bin Fahd University, Al Khobar, Saudi Arabia

**Keywords:** microbial biosynthesis, biocatalysis, cell factory, fermentation, bioprocessing

## Introduction

Recent years have observed a significant surge in research and industrial focus on the biosynthesis of valuable chemicals and natural products. This trend has been primarily driven by the increasing demand for green manufacturing of pharmaceuticals, food ingredients, agricultural products, bioplastics, and important chemicals. Microorganisms play a central role in biosynthesis, and the effectiveness of biocatalysts determines the overall success and acceptance of biosynthesis technologies. With substantial research efforts in strain and bioprocess engineering, there have been significant developments in biosynthesis. Nevertheless, many of the developed technologies for biosynthesis have not been explored commercially due to their low competitiveness and inadequate production performance metrics. The primary challenges include the low stability and tolerance of microorganisms to stressors, slow growth rate, unbalanced metabolic pathways, and inefficient distribution of carbon flux, leading to unwanted byproducts.

Overcoming these challenges requires multidisciplinary and multidimensional efforts, and hence, there has been a growing interest in the exploration of novel strains and metabolic engineering strategies to develop efficient cell factories. These approaches particularly aimed to modulate pathways and engineer strains to enhance the performance of biocatalysts beyond what is observed in their wild counterparts. Furthermore, considerable attention has been devoted to the midstream step, which involves fermentation and requires careful configuration and simulation to achieve the desired levels of titer, yield, and productivity for the target product.

The objective of this Research Topic (RT) was to provide valuable insights into recent advancements in engineering strategies for the development of cell factories and bioprocesses, leading to the establishment of sustainable biosynthesis technologies. With a compilation of 10 articles, this RT offers an up-to-date overview of various aspects, including microbial hosts as cell factories, metabolic pathways, product streams, bioprocessing strategies, and optimization techniques used in biosynthesis. The published articles were authored by researchers affiliated with esteemed institutions across the globe, including countries like the United States, China, Denmark, Sweden, South Korea, Colombia, and Spain. The editorial team expresses sincere gratitude to all the authors for their valuable contributions to this RT.

## Biocatalysts and microbial hosts as cell factories

Biosynthesis of value-added chemicals primarily involves biocatalysts, which are generally categorized into enzymatic and microbial biocatalysts. In a study, a novel enzymatic biocatalyst was developed for D-phenyllactic acid biosynthesis by immobilizing lactate dehydrogenase with Fe_3_O_4_ nanoparticles on a metal–organic framework (Sun et al.). However, microbial systems are generally more convenient and cost-effective compared to biocatalysts. Microorganisms play a central role in biosynthesis, where a wide range of hosts have been explored. The selection of a suitable host depends on factors such as feasibility, genomic knowledge, available engineering tools, and the intended use of the target product. For instance, the model host *E. coli* is frequently used in target product biosynthesis due to its extensive genetic information and engineering capabilities, as demonstrated in Carranza-Saavedra et al. Nevertheless, if the synthesized product is intended for use in food preparations, the use of *E. coli* as a cell factory may raise safety concerns. In such cases, attention may shift to generally recognized as safe (GRAS) strains, as reported in a study by using *Bacillus subtilis* for 3-hydroxypropionic acid (3-HP) biosynthesis (Garg et al.).

The development of a cell factory for the target host requires the construction of a pathway, which can be native or heterologous, and may consist of either product-specific or general pathways. For example, a *B. subtilis* cell factory containing the 3-HP producing pathway was used to biosynthesize 3-HP from glycerol (Garg et al.), while a general pathway like the glyoxylate cycle (GOC) is capable of producing various value-added compounds. This RT published a review on GOC that comprehensively discussed the metabolic regulation of GOC and advancements in biosynthesis performed with this pathway (Yang et al.).

## Product streams

To date, a wide range of value-added and platform chemicals have been reported to be produced by microbial biosynthesis from inexpensive and non-fossil fuel-based substrates with competitive titer and productivity. For example, caproate (hexanoate), which is a valuable platform chemical with its applications in various fields, was biosynthesized from sugars (Otten et al.). Using clostridial cell factories, the authors reported up to 200 mM titer and 8.1 mM/h productivity of caproate. Likewise, biosynthesis of several other platform chemicals was also reported in this RT, including 3-HP (Garg et al.) and 2-ketoisovalerate (Carranza-Saavedra et al.). Apart from platform chemicals, the production of enzymes has also gained attention. For example, alkaline protease, which has widespread industrial applications, was successfully produced by microorganisms heterologously expressing a relevant gene from *B. subtilis* into *B. amyloliquefaciens* (Jiang et al.). Similarly, laccase production has been reported using *Trametes versicolor* and a novel inducer called copper-glycyl-L-histidyl-L-lysine (GHK-Cu) (Wang et al.). Another group of products is dedicated to food or health-related applications, and this RT has reported biosynthesis of several such products, including adenine (Sun et al.), D-phenyllactic acid (Sun et al.), and monoclonal antibodies (Liang et al.).

## Engineering cell factories and bioprocesses

Recent advancements in microbial biosynthesis have utilized advanced technologies to obtain efficient microbial systems and bioprocesses. Coculture technology is one of them that has received significant attention recently. In one study, a novel coculture system was designed for caproate biosynthesis using clostridial species, specifically *Clostridium kluyveri* and *C. saccharolyticum* (Otten et al.). This system exhibited notably higher titers and productivity than monoculture systems. Once a cell factory is developed, the next step is bioprocessing of appropriate substrates, typically involving fermentation. However, this technology presents certain challenges, such as the non-targeted flow of carbon flux. Additionally, achieving optimal efficiency during fermentation can be difficult due to varying conditions between cell growth and product synthesis. In this context, resting cell or whole cell technology has gained attention in recent years. This technology was applied in a study on salidroside biosynthesis, which is a plant-derived bioactive compound (Yang et al.). The authors optimized the conditions and reported excellent conversion efficiency and regioselectivity using the whole cells of *Aspergillus oryzae*. In another attempt, chemical and enzymatic synthesis methods were replaced for adenine by exploring fermentation and enzymatic approaches. A fermentation system coupled with a ceramic membrane was employed to enhance permeability (Sun et al.). This technology led to significant improvements in cell viability and product recovery, with an average adenine titer of 14 g/L.

## Conclusion and outlook

Over the past two decades, there has been a significant research focus on the biosynthesis of valuable compounds with microorganisms playing a crucial role. The aim has been to develop efficient cell factories and bioprocess technologies for sustainable biosynthesis. This RT presents a compilation of 10 high-quality articles, showcasing various research efforts. These articles cover essential aspects such as cell factories, host streams, product ranges, and emerging technological strategies. However, to fully unlock the potential of these biosynthesis technologies, it is imperative to address the challenges associated with microorganisms and bioprocessing through further innovative research. By doing so, we can pave the way for the widespread adoption of biosynthesis and drive the development of sustainable and efficient production methods across a wide range of applications.

